# Camera trap data suggest uneven predation risk across vegetation types in a mixed farmland landscape

**DOI:** 10.1002/ece3.9027

**Published:** 2022-07-13

**Authors:** Amelie Laux, Matthias Waltert, Eckhard Gottschalk

**Affiliations:** ^1^ Department of Conservation Biology University of Göttingen Göttingen Germany

**Keywords:** camera traps, farmland, ground‐nesting farmland birds, *Perdix perdix*, predation risk, vegetation type

## Abstract

Ground‐nesting farmland birds such as the grey partridge (*Perdix perdix*) have been rapidly declining due to a combination of habitat loss, food shortage, and predation. Predator activity is the least understood factor, especially its modulation by landscape composition and complexity. An important question is whether agri‐environment schemes such as flower strips are potentially useful for reducing predation risk, for example, from red fox (*Vulpes vulpes*). We employed 120 camera traps for two summers in an agricultural landscape in Central Germany to record predator activity (i.e., the number of predator captures) as a proxy for predation risk and used generalized linear mixed models (GLMMs) to investigate how the surrounding landscape affects predator activity in different vegetation types (flower strips, hedges, field margins, winter cereal, and rapeseed fields). Additionally, we used 48 cameras to study the distribution of predator captures within flower strips. Vegetation type was the most important factor determining the number of predator captures and capture rates in flower strips were lower than in hedges or field margins. Red fox capture rates were the highest of all predators in every vegetation type, confirming their importance as a predator for ground‐nesting birds. The number of fox captures increased with woodland area and decreased with structural richness and distance to settlements. In flower strips, capture rates in the center were approximately 9 times lower than at the edge. We conclude that the optimal landscape for ground‐nesting farmland birds seems to be open farmland with broad extensive vegetation elements and a high structural richness. Broad flower blocks provide valuable, comparatively safe nesting habitats, and the predation risk can further be minimized by placing them away from woods and settlements. Our results suggest that adequate landscape management may reduce predation pressure.

## Graphical abstract

Red fox in a flower strip. Credit: Amelie Laux.

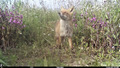

## 1 INTRODUCTION

Agricultural landscapes cover large areas (e.g., 45% in the EU, 46% in the USA [Bigelow & Borchers, 2017; EC, 2018]) and harbor an important part of terrestrial biodiversity (Krebs et al., 1999; Robinson et al., 2001). In the last decades, agro‐biodiversity has been decreasing rapidly and many farmland bird species have exhibited drastic population declines (Burns et al., 2021; Kamp et al., 2021). Negative effects of agricultural intensification are the main drivers of these declines, in particular habitat loss due to an increase in field sizes and monocultures and food scarcity due to the increasing usage of pesticides and fertilizers (Donald et al., 2001, 2006; Gibbons et al., 2015; Newton, 2004; Pickett & Siriwardena, 2011). For example, the pesticide‐induced lack of insects increases the mortality of grey partridge *Perdix perdix* chicks, which depend on insect‐food in their first 2 weeks of life (Potts & Aebischer, 1995).

Predation is the second major reason for farmland bird declines, especially in ground‐nesting birds such as grey partridge *Perdix perdix*, lapwing *Vanellus vanellus* or skylark *Alauda arvensis* (Donald et al., 2002; Macdonald & Bolton, 2008; Potts & Aebischer, 1995; Roos et al., 2018). Many studies have identified mammals such as red foxes *Vulpes vulpes* or mustelids as the main predators of ground‐nesting farmland birds (Bro et al., 2000; Gottschalk & Beeke, 2014; Langgemach & Bellebaum, 2005; Macdonald & Bolton, 2008; Morris & Gilroy, 2008; Potts, 2012; Roos et al., 2018). Avian predators, principally corvids and raptors, play a smaller role in general, although some studies found substantial nest predation by corvids (Arbeiter & Franke, 2018; Bravo et al., 2020; Bro et al., 2000; Draycott et al., 2008; Faria et al., 2022; Krüger et al., 2018; Macdonald & Bolton, 2008; Stoate & Szczur, 2001). Corvids usually predate eggs or small chicks, while foxes and other mammals frequently prey on adult birds as well, in particular on incubating hens (Bro et al., 2000; Draycott et al., 2008; Gottschalk & Beeke, 2014; Potts, 2012). Hence, mammalian predators likely have a higher negative impact on ground‐nesting farmland bird populations than avian predators.

Predator numbers in Europe have been increasing in recent decades following the successful anti‐rabies vaccination of foxes and badgers *Meles meles*, decreasing hunting pressure, and the expansion of new predator species such as racoon *Procyon lotor* and racoon dog *Nyctereutes procyonoides* (Bartoszewicz, 2011; Beltrán‐Beck et al., 2012; Chautan et al., 2000; Griffiths & Thomas, 1993; Kauhala & Kowalczyk, 2011; Keuling et al., 2011; Kowalczyk, 2014). However, increasing predator numbers account only partly for the increase in predation pressure. Changes in land use and landscape composition due to agricultural intensification also play a key role (Evans, 2004; Whittingham & Evans, 2004). Habitat loss can cause birds to nest in sub‐optimal, exposed sites or to congregate in the few remaining habitat patches, which also are highly attractive for predators (Evans, 2004; Panek & Kamieniarz, 2000; Whittingham & Evans, 2004). Bad habitat conditions can further limit the possibility to compensate predation losses by rearing additional broods (Whittingham & Evans, 2004). A study in France found that impoverished landscapes can drive partridges into riskier areas, for example in close proximity to woods, settlements, and roads (Harmange et al., 2019). In Poland, predation rates of grey partridges by foxes were higher in homogenous landscapes than in richly structured landscapes (Panek, 2013). In that study, fox activity in homogenous landscapes was concentrated in scarce permanent vegetation, which was also the preferred nesting habitat of partridges. In heterogeneous landscapes with a high number of hedges and other permanent vegetation, fox activity was distributed among a larger area and thus the encounter probability between partridges and foxes was lower (Panek, 2013).

Ongoing population declines in many ground‐nesting farmland birds demonstrate that current conservation measures are not sufficient to maintain populations (Fox, 2004; Heldbjerg et al., 2018). While habitat loss and food scarcity can be, at least partly, compensated by dedicated set‐asides, flower strips, and other habitat improvements (Gottschalk & Beeke, 2014; Potts, 2012; Rands, 1986), high predation pressure remains a problem and may prevent population growth (Newton, 1998; Roos et al., 2018). Even predator presence alone (i.e., without a predation attempt) can cause disturbances and can have sublethal effects on ground‐nesting birds (Cresswell, 2008; Cresswell & Quinn, 2013).

Different strategies have been proposed to reduce predation pressure (Doherty & Ritchie, 2017; Laidlaw et al., 2021; Roos et al., 2018). Lethal predator control is the most widespread intervention (Ewald et al., 2012; Reynolds et al., 2010; Tapper et al., 1996; White et al., 2014), but several studies suggest that predator control is difficult to implement effectively at the landscape level and often presents ethical problems (Rushton et al., 2006; Bolton et al., 2007; Lieury et al., 2015; Doherty & Ritchie, 2017; Kämmerle, Niekrenz, et al., 2019; Kämmerle, Ritchie, et al., 2019; Laidlaw et al., 2021). Habitat management may offer an alternative approach (Laidlaw et al., 2015, 2017). If we understand how predators use the landscape and where their activity, and thus the predation risk, is highest, we may be able to manage the landscape in a way that improves habitat quality and minimizes predation risk (Doherty & Ritchie, 2017; Evans, 2004; Laidlaw et al., 2021; Langgemach & Bellebaum, 2005; Roos et al., 2018).

At present, there are many open questions regarding the effect of landscape composition on predator activity and its implications for farmland bird conservation. How do landscape features such as forests, settlements, and water bodies influence predator activity? Can narrow, linear structures act as ecological traps (Eglington et al., 2009; Rantanen et al., 2010; Suvorov & Svobodová, 2012)? Are landscapes with a lot of hedgerows more risky for ground‐nesting birds? Or do more structures lead to a better distribution of predator activity and thus decrease predation risk?

In this study, we investigate how predation risk by mammals is mediated by landscape composition. Grey partridges were the conservation target of this study, but the results could be equally valuable for other ground‐nesting farmland birds and many species affected by high predation rates.

We ask (i) Which are the main predators in farmland? (ii) Are there differences in predator activity between vegetation types? (iii) Which environmental parameters explain spatial variation in predator activity best? And (iv) How do predators use flower strips, one of the most popular farmland conservation measures?

## 2 METHODS

### 2.1 Data collection

#### 2.1.1 Study area

**FIGURE 1:**
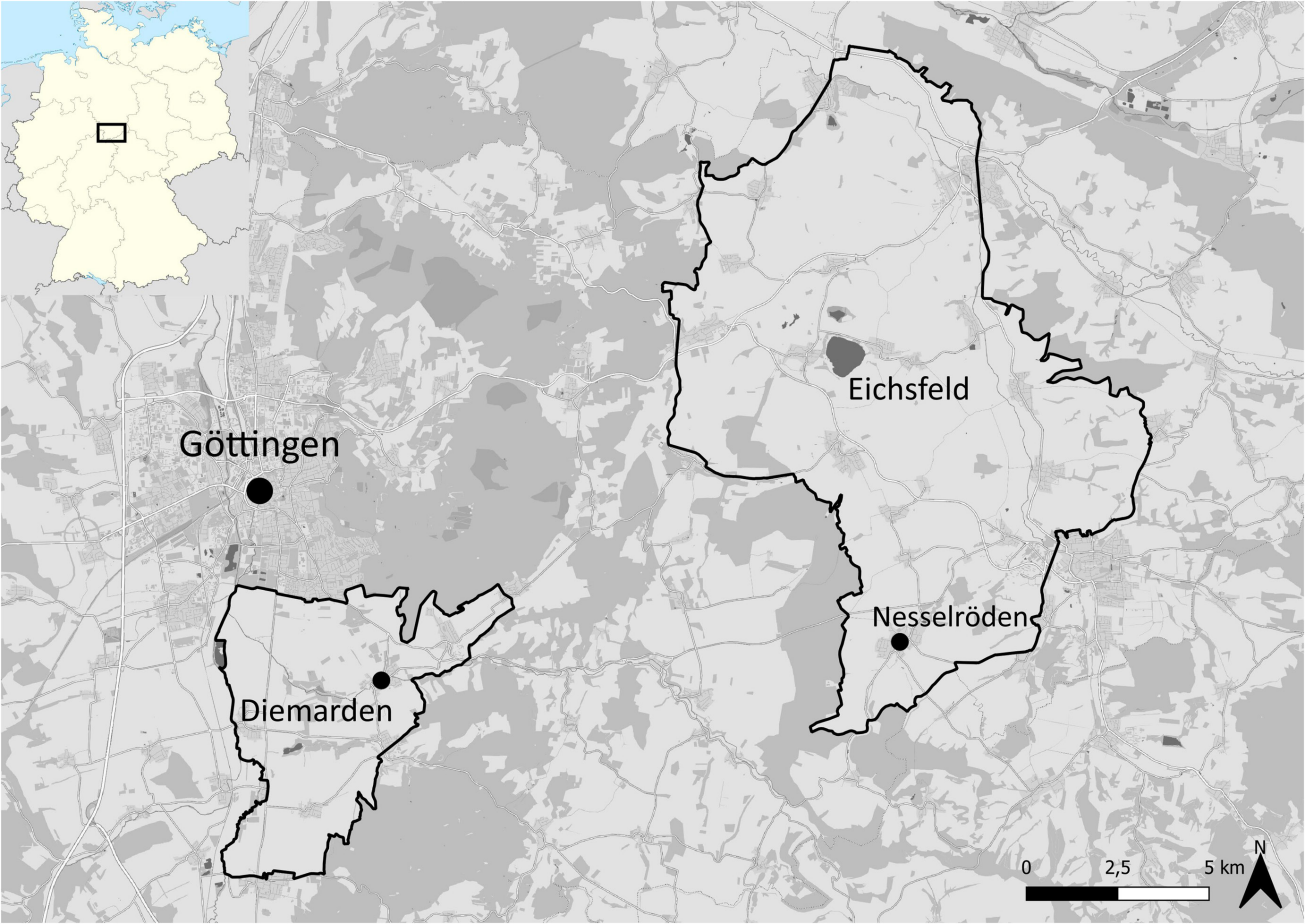
Map of the study area with the villages Diemarden and Nesselröden (CartoDB, 2021; NordNordWest, 2008; QGIS Development Team, 2021)

The study area was located near Göttingen in Lower Saxony, Germany, and was based on the area covered by already existing partridge telemetry data to encompass the main partridge distribution in the district (Figure 1). One part of the study area, “Diemarden,” lay directly south of Göttingen and covered 35 km^2^. The other part, “Eichsfeld,” was located east of Göttingen and encompassed 131 km^2^. Both areas have a comparable landscape structure—they are hilly semi‐open cultural landscapes dominated by agriculture and small villages (Diemarden: 83% arable, 7% grassland, 6.9% settlements, Eichsfeld: 73% arable, 12% grassland, 8.56% settlements [LGLN, 2019; TLBG, 2019]) Large forests were excluded from the study area, therefore forest cover is only 1.9% in “Diemarden” and 3.6% in “Eichsfeld,” although both areas are bordered by extensive forests.

#### 2.1.2 Predator activity as a proxy for predation risk

We used predator activity as a proxy for predation risk because the predation risk posed by different predators for ground‐nesting birds is difficult to measure directly. Activity was measured as the number of predator captures at each camera site. We assumed that a higher predator activity corresponded with a higher predation risk.

#### 2.1.3 Vegetation types

We focused on five vegetation types that were found to be important to grey partridges in spring and summer according to telemetry studies by Gottschalk and Beeke (2014): flower strips, field margins, hedges, winter cereal fields, and rapeseed fields. All flower strips in this study were “structurally rich flower strips,” where one half of each flower strip is resown every year to create a mix of annual and perennial vegetation (“strukturreiche Blühstreifen” AUM BS12, Nds. Ministerium für Ernährung, Landwirtschaft und Verbraucherschutz, 2022). Flower strips were variable in width, from a minimum width of 6 m to extensive flowering areas. Field margins were grassy margins along the edge of fields. All hedges had a minimum length of 10 m and were at least 3 m wide.

#### 2.1.4 Camera traps

Browning Strike Pro HD camera traps (HDPX‐5, Browning Trail Cameras) were used to record predators. They were mounted on wooden stakes approximately 40 cm above the ground and placed either in the center of the field or flower strip, or, for the vegetation type “field margin,” on the border between field and field margin. In hedges, cameras were placed inside of the hedge wherever possible and next to the hedge otherwise. No bait was used, but cameras were placed along tractor lanes or animal paths to ensure a similar field of view. Cameras were set to take two sequential pictures once triggered to facilitate species identification.

#### 2.1.5 Sampling design

##### Predator activity within the landscape

In the main survey, we used 120 camera stations that were evenly stratified between the five vegetation types (i.e., 24 cameras were placed in each vegetation type). The number of camera stations allocated to each of the two study areas was proportional to the available amount of each vegetation type. The camera sites themselves were distributed randomly. For this purpose, a 500 m × 500 m grid was overlaid over each study area and the grid cells for each vegetation type were chosen randomly. Only grid cells that were at least 50% inside the study area and had a maximum of 50% forest or settlement cover were considered and only one camera was allowed per grid cell. Within a grid cell, we selected the available field (flower strip, hedge, field margin) that was closest to the center of the grid cell. Permission to install cameras was obtained from each farmer and game tenant.

Data sampling took place in 2019 and 2020 between May and July to align with the breeding season of grey partridges. Camera sites remained the same between years, except where winter cereal, rapeseed, or flower strip sites had to be changed due to crop rotation. In these cases, the nearest suitable and available field was selected as replacement. Due to logistical constraints, only 40 sites could be sampled simultaneously. Therefore, we created three time blocks and cameras were rotated after each time block. In each time block, eight sites were chosen at random for each vegetation type. Cameras were in operation for at least 20 full days (max. 27 days). Cameras with less than 15 continuous sampling days were repeated once, either in the next time block or in a fourth time block at the end of the season. We only analyzed data collected during the longer sampling period.

##### Predator activity in flower strips

We complemented our main survey by studying, how predation risk is distributed in flower strips, namely, the differences between the edges and the interior of flower strips. Twenty‐four randomly selected flower strips were sampled in August 2020, 12 in each part of the study area. The flower strips were located around the villages of Diemarden and Nesselröden, respectively (see Figure 1). These areas were part of the Interreg Partridge Project (PARTRIDGE, 2022) and were chosen for easy access to the flower strips. In each flower strip, two cameras were placed simultaneously, one at the edge and one directly opposite 10 m inside of the flower strip. The inside camera was placed 10 m from the edge regardless of vegetation density, but an area of approximately 1 m^2^ was cleared to allow visibility. The cameras at the edge had a larger field of view, but we included only predators that passed within 1 m of the camera in our analysis to ensure comparability across sampling locations. Cameras were in operation for 20–22 full days and they were checked once after 9–10 days to change SD‐cards if necessary.

### 2.2 Picture analysis

Pictures were sorted with Digikam 6.1.0 (digiKam, 2019) and all predators were identified to species level. Stone marten *Martes foina* and pine marten *Martes martes* were summarized as “marten” and domestic cats *Felis catus* and wildcats *Felis silvestris* were summarized as “cats,” because identification to species level was not always possible. Wild boars *Sus scrofa* were considered predators for the purpose of this study as they frequently predate ground‐nesting bird nests (Barrios‐Garcia & Ballari, 2012). Consecutive records of the same species at the same site had to be at least 10 min apart to be considered independent captures, except when individuals could be identified. Multiple animals in the same picture were counted separately.

### 2.3 Statistical analysis

All analysis were carried out using R version 4.1.3 (R Core Team, 2021) and figures were plotted using ggplot2 (Wickham, 2016) and ggeffects (Lüdecke, Aust, et al., 2021). Because our data were not normally distributed (Shapiro–Wilk Test, all *p* < .001, Table A1), non‐parametric tests were used where applicable.

We combined data from both parts of the study area for our analyses. Several reasons motivated this choice: (a) both parts of the study area are very close together compared to their size and very similar in landscape composition, therefore we do not expect predator activity and predator's responses to environmental parameters to vary between areas, (b) we are interested in the effects of environmental predictors on predator activity, and those predictors should capture and explain any differences between the two areas, (c) a Wilcoxon rank sum test (R‐package “stats”, R Core Team, 2021) showed no significant differences between the activity indices of free‐ranging predators (i.e., excluding dogs) in both areas (all *p* > .05, Table A2).

**FIGURE 2:**
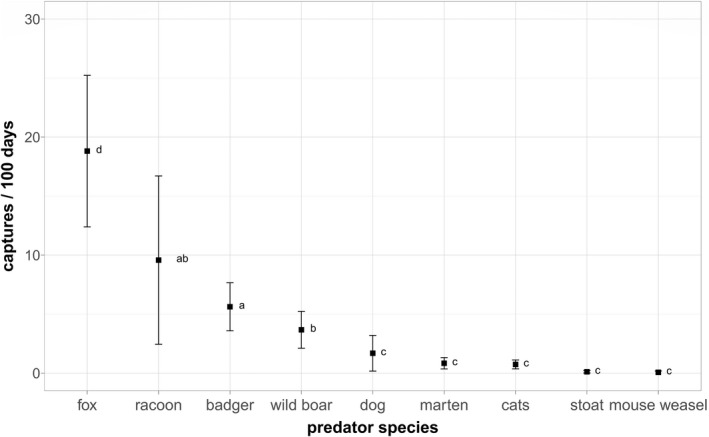
Mean capture rates (captures/100 days) for each predator in all vegetation types. *N*_sites_ = 240, 2019 and 2020 together. Kruskal–Wallis chi squared = 543.64, df = 8, *p* < .001 (Table A8). Letters correspond to significant differences following a post‐hoc Dunn's test (Table A9)

For completeness, the mean capture rate of domestic dogs *Canis lupus familiaris* is shown in Figure 2 (see Section 3). We excluded domestic dogs from all further analyses, however, because the number of dog captures depends on human behavior (e.g., popular walking routes or proximity to car parks) rather than the dog's habitat selection.

#### 2.3.1 Comparison of predator capture rates and vegetation types

To enable comparisons between sites with different sampling times, the number of observations per species was standardized as the capture rate per 100 camera days for each camera. To determine which predator species was the most prevalent, we compared capture rates between species for all camera sites and separately for each vegetation type.

Similarly, we compared capture rates between vegetation types. To compare overall predator activity, we calculated the capture rate for all predator species except dogs together, hereafter “all predators,” and compared that between vegetation types. We also compared fox capture rates between vegetation types, as foxes were revealed to be the most frequently observed predators (see Section 3).

Kruskal–Wallis rank sum tests (R‐package “stats”, R Core Team, 2021) were used for all comparisons and followed by Dunn's Post‐Hoc tests with Holm's procedure to adjust p‐values for multiple comparisons, if the former were significant (R‐package “FSA” 0.9.2, Ogle et al., 2021). All comparisons were calculated based on the combined data for 2019 and 2020, because Wilcoxon rank sum tests (R‐package “stats”, R Core Team, 2021) found no significant differences between the years for any species or vegetation type (all *p* > .05, Table A3).

#### 2.3.2 Model set M1: Detailed models for predator and fox activity in summer

We used generalized linear mixed models (GLMMs) to analyze the effects of landscape composition and vegetation type on the number of total predator captures and fox captures separately. We focused on foxes in addition to “all predators” because they were by far the most prevalent predator species in our study (see Section 3) and are widely considered to be one of the most important predators for partridges and other ground‐nesting birds (Langgemach & Bellebaum, 2005; Potts, 2012; Reynolds & Tapper, 1995; Roos et al., 2018).

For these models, we generated detailed landscape composition metrics within a buffer of 500 m around each cameras (see Section 2.3.2.1 below, Table 1). In addition, we performed a sensitivity analysis regarding the spatial scale at which predictors were measured by comparing three GLMMs based on predictors measured in 500 m, 1 km, and 2.5 km buffers around the camera sites, respectively. The results confirmed that landscape composition at the local scale (500 m) was most important (see Appendix B for methods and results of this comparison; Tables B1, B2, B3, B4, B5, B6).

**TABLE 1:** List of predictors considered in the analysis of predator and fox activity in model set 1

	Predictor	Explanation	Unit	Source
Distances	Wood_Dist	Distance to next wood, including hedges, small woods and forests	m	B‐DLM, our maps
	Water_Dist	Distance to next running or standing water	m	B‐DLM
	Settl_Dist	Distance to next settlement	m	B‐DLM
	Edge_Dist	Distance to next field edge	m	InVeKos, our maps
	Road_Dist	Distance to next road outside of settlements, including railways	m	B‐DLM
Land cover within a 500 m buffer	Wood_Area	Hedges, small woods and forests	ha	B‐DLM, our maps
	Ext_Area	Area of extensively used grassland, fallows, flower strips and similar environmental schemes	ha	InVeKos
	Arable_Area	Area of arable land	ha	InVeKos
	Settl_Area	Area of settlements	ha	B‐DLM
	Water_Area	Surface area of all running and standing water	ha	B‐DLM
	Edge_Area	Area of field margins	ha	Our maps
	Road_Density	Area of roads and railways outside of settlements	ha	B‐DLM
	Border_Length	Length of field block borders	km	InVeKos
	Hab_Div	Shannon‐Index based on land cover types within a 500 m buffer: wood, water, settlement, field margin, crop type	Shannon‐Index	B‐DLM, InVeKos, our maps
Site based	Vegetation type	Vegetation type at camera site: Field margin, flower strip, hedge, rapeseed or winter cereal	factor	–
	Mean_Field	Mean field size of all fields (partly) within the 500 m buffer	ha	InVeKos
	Year	2019 or 2020	factor	–
	Block	Time blocks 1–4 in each year	factor	–
	Run time	Active camera time	min	Empirical

*Note:* Predictors in grey were not used in the full model due to collinearity issues. Vegetation types included in the Shannon Index were woods, water, settlements, field margins, winter cereal, summer cereal, fallow, maize, permanent grassland, winter rapeseed, summer rapeseed, orchards, turnips, short term woods, forage, root crops, protein crop, oilseed crops, pseudocereal, and “others.” Data sources: B‐DLM (LGLN, 2019; TLBG, 2019), InVeKos (SLA, 2019a, 2019b, 2020), our maps.

##### Environmental predictors

Table 1 shows the predictors considered in the analysis of landscape composition effects on predator activity. All predictors were calculated in R 4.1.1 (R‐package “sf” 1.0‐3, Pebesma et al., 2018; R Core Team, 2021) using the Digital Basic‐Landscape Model (LGLN, 2019; TLBG, 2019) for settlements, streets, forests, and water bodies and the 2019 and 2020 InVeKos data for Lower Saxony (SLA, 2019a, 2019b, 2020) for crop types and field borders. We developed our own maps for hedges, small woods, and field edges, for which there were no official maps available. Within a 500 m buffer area around each camera site, all hedges, woods, and field margins were first mapped in QGIS (QGIS Development Team, 2021) based on Google Satellite imagery and later verified in the field.

We assessed the continuous environmental predictors for collinearity by calculating the Variance Inflation Factor (VIF) and sequentially dropped predictors with high VIF—values, until all VIF <3 (“HighstatLibV10.R” Zuur et al., 2009, 2010). The area of arable land (Arable_Area) and road density (Road_Density) were dropped, because they were closely related to the area of woodland and distance to road (Wood_Area and Road_Dist), respectively. Furthermore, we dropped the mean field area (Mean_Field) as it was closely related to the length of field borders (Border_Length) and the area of field edges (Edge_Area) and we were more interested in the effect of field margin structure on predator activity. We assessed collinearity between the selected continuous predictors and the categorical predictor “vegetation type” by calculating the General Variance Inflation Factor (GVIF) and its derivative GVIF^(1/2 df)^, which corresponds to √VIF (Fox & Monette, 1992; “HighstatLibV10” Zuur et al., 2009). GVIF^(1/2 df)^ was below 2 for all predictors (corresponding to a VIF‐value <4, Table A4), suggesting no collinearity in our remaining set of environmental predictors (compare Heringer et al., 2019; Min et al., 2019; Pebsworth et al., 2012; Vega et al., 2010).

##### Study covariates

We used a random effect of time block nested in year to account for variation in predator activity over time. Study site area (i.e., Diemarden or Eichsfeld) was not included as a covariate as there were no significant differences between “all predator” or fox activity between the areas (see Section 2.3).

##### Model formulation

We analyzed predator activity by fitting GLMMs with a negative binomial distribution of errors and the number of captures as the response variable. Akaike's Information Criterion (AICc) corrected for small sample sizes was used for comparisons between models. Separate models were fit for “all predators” and “fox”.

We used a negative binomial distribution, because GLMMs with a Poisson distribution indicated very strong overdispersion and a bad fit to the data. There was no zero‐inflation detected and zero‐inflated negative binomial models showed no improvement in model fit based on AICc. Models were fit using the R package glmmTMB 1.1.2.3 (Brooks et al., 2017) and model fit was examined visually with QQPlots and residual vs fitted plots using the DHARMa package version 0.4.5 (Hartig & Lohse, 2021). Additionally, we verified model assumptions by testing model residuals for homogeneity of variances (Levene's Test) and uniformity (Kolmogorov–Smirnov test) using DHARMa (Hartig & Lohse, 2021). *R* ^2^ was calculated as Nakagawa's *R* ^2^ for mixed models (R‐package “performance” 0.9.0, Lüdecke, Ben‐Shachar, et al., 2021; Lüdecke, Makowski, et al., 2021). Moran's I (Moran, 1950) (R package “ape” 5.6‐2, Paradis & Schliep, 2019) suggested no spatial autocorrelation in the raw data or in the model residuals (Table A5).

Global models included distance to wood, distance to field edge, distance to water, distance to traffic, distance to settlement, wood area, extensive area, field margin, settlement area, water area, length of field borders, habitat diversity, and vegetation type as fixed effects and time block nested into year as random effect. In all models, flower strip was used as the reference level for the factorial covariate vegetation type. The runtime of each camera in minutes was used as offset to correct for sampling periods of different length.

We used backward selection based on AICc on the fixed effects to select the most parsimonious models. Starting with the global model, each fixed effect was dropped in turn and the AICc of the reduced model calculated. The fixed effect that caused the largest reduction in AICc was dropped permanently and the procedure repeated until no further reduction in AICc occurred.

##### Relative variable importance

For each final model, we analyzed the relative importance of variables through a random permutation procedure. We randomized each variable in turn and calculated the correlation between the predictions made by the randomized and original models (Thuiller et al., 2009). This procedure was repeated 100 times for each variable. Next, we calculated the importance value for each variable as one minus the mean correlation between the predictions made by the original and randomized models and standardized the relative importance value to one (Thuiller et al., 2009).

#### 2.3.3 Predator and fox activity in and around flower strips

As before, the number of observations per species was standardized as the capture rate per 100 camera days to enable comparisons between sites with different sampling times. We used Wilcoxon signed rank tests with continuity correction (R‐package “stats”, R Core Team, 2021) to compare fox and total predator capture rates between edge‐cameras and inside‐cameras in flower strips. All flower strips from Diemarden and Nesselröden were analyzed together, because a Wilcoxon rank sum test (R‐package “stats”, R Core Team, 2021) showed no significant differences between the capture rates of either “all predators” or foxes in both areas (Table A15). A Kruskal–Wallis test (R‐package “stats”, R Core Team, 2021) followed by a Dunn's Post‐Hoc Test with Holm's procedure to adjust p‐values for multiple comparisons (R‐package “FSA” 0.9.2, Ogle et al., 2021) was used to compare capture rates between predator species at each position.

## 3 RESULTS

Overall, our main survey yielded 2122 camera trap observations of predators from 5024.697 active camera days over 2 years in summer 2019 and summer 2020. Over both years, depending on vegetation type, between 41.67% (in winter cereal) and 95.83% (in rapeseed) of all cameras recorded at least one predator (Table A6). In flower strips, 79.17% of the cameras recorded predators (Table A6). The following predators were captured: fox, racoon, badger, wild boar, marten, cats, stoat *Mustela erminae*, mouse weasel *Mustela nivalis*, and dogs.

In addition, we analyzed 236 predator observations from 855.409 active camera days recorded at the edge or in the center of flower strips in the second survey. Predators were recorded by 95.83% of all the cameras at the edge of flower strips and by 54.17% of the cameras in the center of flower strips.

### 3.1 Comparison of predators

Figure 2 shows the mean capture rates at all camera stations for each predator species. Foxes were captured significantly more frequently than any other predator species (mean 18.82 captures/100 days, standard deviation [SD] 50.6; Tables A7–A9). If the vegetation types were analyzed individually, foxes were the most frequent predator in every vegetation type except for hedges and rapeseed fields, where there was no significant difference compared to racoons (Table A9).

### 3.2 Comparison of vegetation types

**FIGURE 3:**
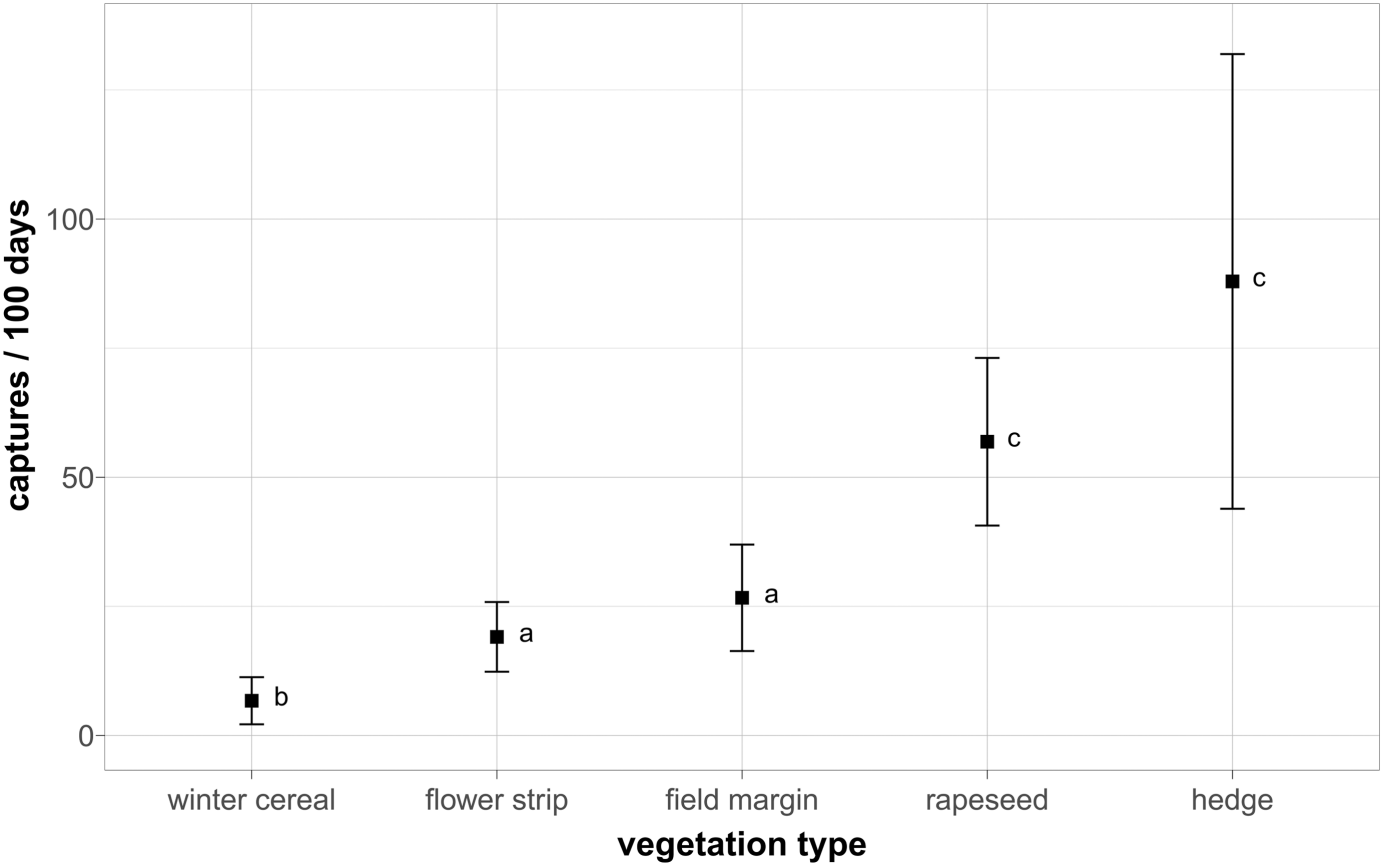
Mean capture rate (captures/100 days) of “all predators” in different vegetation types. *N*_sites_ = 240. Kruskal–Wallis chi squared = 78.26, df = 4, *p* < .001 (Table A10). Letters correspond to significant differences following a post‐hoc Dunn's test (Table A11)

Figure 3 shows the mean capture rates in different vegetation types for all predator species together, except dogs (see Section 2.3). The number of predator captures in flower strips (mean 19.09 SD 23.23) was significantly lower than in hedges (mean 87.93, SD 151.62) and rapeseed fields (mean 56.88, SD 55.88) and also less than in field margins, although this difference was not significant (Tables A7, A10, A11). A similar pattern between vegetation types was observed for foxes, although only the differences between winter cereal and the other vegetation types were significant (Tables A7, A10, A11).

### 3.3 Model set M1: Detailed models for the number of predator and fox captures in summer

We modeled the effects of various environmental parameters on fox and “all predator” activity, as measured by the number of captures. Both models yielded very similar results, most likely because foxes were the main predator in our study and responsible for most predator captures. Therefore, we show only the results for fox captures in detail in this section. Results for “all predator” captures can be found in Appendix A (Tables A12 and A13).

#### 3.3.1 Number of fox captures

**FIGURE 4:**
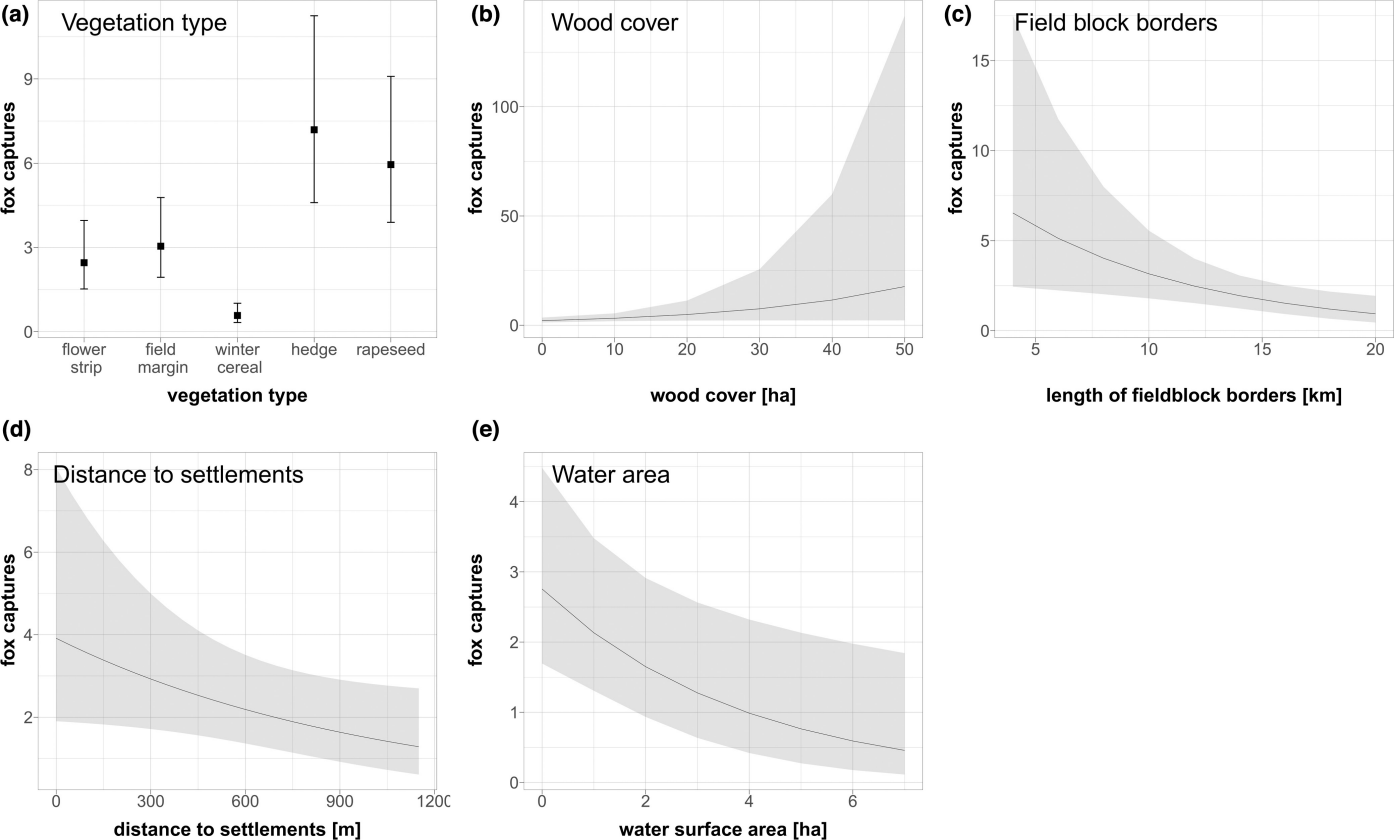
Plots of generalized linear mixed model “M1 fox activity” describing the effects of environmental parameters on the number of fox captures. Significant variables: Vegetation type, water area, field block borders (Table 2)

Water area, distance to settlements, length of field block borders, wood area, and vegetation type were retained as important explanatory parameters for the number of fox captures after backward selection (Table 2; full model results in Table A14). Fox captures decreased significantly with increasing water area and increasing length of field borders. Fox captures also decreased marginally significantly with increasing distance to settlements and increased marginally significantly with increasing wood area. Additionally, the relationship between the number of fox captures and vegetation type was significant. Compared to flower strips, fox captures decreased significantly in winter cereal and significantly increased in hedges. Fox captures also increased in field margins and rapeseed fields, but these relationships were not significant. Vegetation type had the highest explanatory power (44.75%), followed by wood area (20.93%) and length of field borders (19.40%) (Table 2, Figure 4).

**TABLE 2:** Model results of M1 Fox activity after backward selection

Predictors		Estimates	SE	*z*‐Value	*p*‐Value	Odds ratio	Relative importance
*Fixed effects*							
Intercept		−7.422	0.81	−9.111	<.001		
Water_Area		−0.257	0.102	−2.513	.012	0.774	7.683
Settl_Dist		−0.001	0.000	−1.95	.051	0.999	7.228
Border_Length		−0.121	0.046	−2.648	.008	0.886	19.402
Wood_Area		0.043	0.023	1.881	.06	1.044	20.933
Vegetation	Field margin	0.214	0.341	0.627	.531	1.239	Vegetation type 44.754
	Winter cereal	−1.448	0.395	−3.664	<.001	0.235	
	Hedge	1.073	0.33	3.251	.001	2.925	
	Rapeseed	0.884	0.321	2.756	.006	2.412	

	Variance	SD	Groups	*N*_Observations_
*Random effects*				
Year:Block	0.005	0.071	8	240

*Note:* Negative binomial generalized linear mixed model. For variable abbreviations see Table 1. AICc = 1069.153, Conditional *R* ^2^ = 0.428, Marginal *R* ^2^ = 0.425. Dispersion parameter = 0.515.

Abbreviations: SE, standard error; SD, standard deviation.

### 3.4 Predator and fox capture rates within and at the edge of flower strips

**FIGURE 5:**
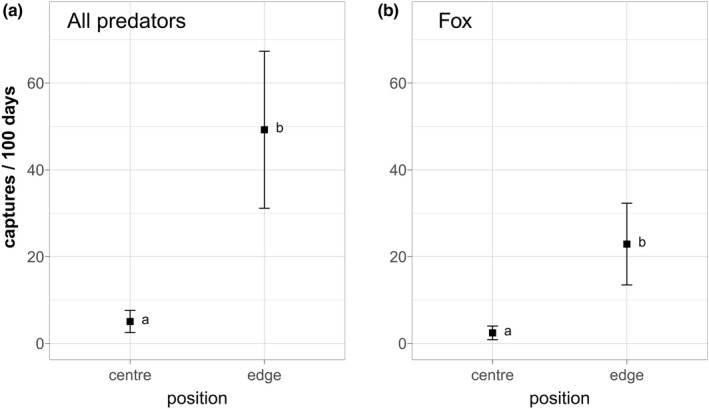
Mean capture rates (captures/100 days) of “all predators” and fox at the edge and in the center of flower strips. *N*_Cameras_ = 24 at each position. Wilcoxon Signed Rank Test: “all predators”: *V* = 13, *p* < .001, fox *V* = 15, *p* < .001 (Tables A16 and A19)

Figure 5 shows the mean capture rates of “all predators” and foxes in the center and at the edge of flower strips. For the edge capture rates, only predators that passed directly by the camera were included to avoid bias due to a larger field of view. In both cases, capture rates were very low in the center (Figure 5; “all predators”: mean 5.06, SD 6.05, fox: mean 2.45, SD 3.70; Tables A16 and A19) and significantly higher at the edge (Figure 5; “all predators”: mean 49.24, SD 42.84, fox: mean 22.9, SD 22.3; Tables A16 and A19). At both positions, fox captures were significantly more frequent than any other predator species (Tables A17 and A18). If all predator captures by edge cameras were included regardless of the distance to the camera, capture rates at the edges increased by 20%–30% and were comparable to the capture rates measured in rapeseed fields and hedges in the main survey (all edge captures: “all predators” mean 60.99, SD 53.31, fox: mean 31.47, SD 34.53; Table A16, compare Table A7).

## 4 DISCUSSION

Our study showed how risky farmland is for ground‐nesting birds. Of 240 cameras, 78.75% recorded at least one predator capture in 20 days. For comparison, grey partridges need around 40 days for laying and incubating a clutch (Cramp, 1980). Red fox activity was significantly higher than that of any other species, accounting for approximately 45% of all observations, which corroborates their importance as predators for ground‐nesting birds (Potts, 2012; Reynolds & Tapper, 1995; Roos et al., 2018). Fox activity appeared to be driven primarily by the vegetation type of the camera site, with wood cover, field borders, distance to settlements, and water surface area playing a smaller role.

The presumably “safest” places in farmland (i.e., those that had the least amount of predator captures) were winter cereal fields, whereas rapeseed fields had a high number of predator captures. Rapeseed fields in summer provide good cover and can support high rodent populations (Heroldová et al., 2011), while the dense winter cereals may make prey less accessible and these fields less attractive to predators. However, in many areas partridges have a strong preference for permanent vegetation such as fallows, margins, and hedges as nesting habitat (Buner et al., 2005; Gottschalk & Beeke, 2014; Potts, 2012). Both the number of fox captures and total predator captures were lower in flower strips than in field margins or hedges, suggesting less predator activity and a lower predation risk in flower strips. This further supports the use of flower strips as highly effective conservation measures for ground‐nesting farmland birds as they can provide safer nesting sites compared to other permanent vegetation structures. In contrast to mostly broad flower strips, hedges, and field margins form linear structures that many predators prefer for orientation, traveling, and hunting, which can explain the higher predator activity in these structures (Andrén, 1995; Bider, 1968; Bischof et al., 2019; Lidicker, 1999; Panek, 2013).

A closer look at predator activity in flower strips also revealed more than nine times as much predator activity along the edges than in the center, where only very few predators were captured. This suggests that predator activity within broad flower strips is much lower than in the surrounding area, presumably because the denser vegetation increases spatial resistance and many predators choose the easier path along the edge (Andrén, 1995; Bischof et al., 2019; Lidicker, 1999). These findings corroborate results from Bro et al. (2000), who found higher predation rates of grey partridges in linear structures, and Gottschalk and Beeke (2014), who showed that nest losses of grey partridges in vegetation structures less than 10 m wide were twice as high as in broader vegetation structures. If the majority of predators move along the edges, the risk of detection and predation is higher in narrow structures and close to the edge. Thus, selection of microhabitats within one habitat type has a large impact on predation risk and the safety of flower strips depends on their shape and size. Broad flower blocks are important to provide safe nest sites.

We found that fox activity was lower in richly structured landscapes, as the number of fox captures was negatively related to field block border length as a measure for structural richness. The number of total predator captures showed a similar negative relation with field margin area (Table A13). Highly structured landscapes may have a lower predation risk due to a “dilution effect,” whereby predators are more widely dispersed among available structures, decreasing the probability of encountering a predator at any given site. Additionally, a structurally rich landscape can offer more suitable nest sites and prevent birds from clustering together in unsuitable or isolated vegetation patches, thereby further reducing predation risk. Similar explanations for this pattern have been proposed by others, for example, Evans (2004) and Whittingham and Evans (2004). Our results also align with those of Panek (2013) who found a higher encounter probability of partridges and foxes in homogenous landscapes with few hedges compared to heterogeneous landscapes. Similarly, Kuehl and Clark (2002) found that the length of strip habitat (i.e., road ditches and fences) was negatively related to the presence of foxes and raccoons. The “all predator model” further showed a positive effect of habitat diversity (Table A13), suggesting that increasing habitat diversity can increase predator activity and thereby predation risk. This is likely due to diverse landscapes supporting larger and more diverse predator communities (Pita et al., 2009; Tews et al., 2004). Yet, our results indicate that this effect may be at least partially mitigated by highly structured landscapes with a large amount of edge structures, which have been shown to reduce the encounter probability between predator and prey. The Shannon Index that we used to measure habitat diversity cannot differentiate between different field sizes and landscapes with the same Shannon Index value could still be widely different in their structure. Additionally, the final fox model did not include habitat diversity, which further indicates that predation risk is affected more by landscape structure than habitat diversity.

We found wood cover to be positively related to fox captures, similar to previous studies (Jankowiak et al., 2008; Keuling et al., 2011; Kuehl & Clark, 2002; Weber & Meia, 1996). Hedges, woods, and forests can be highly attractive for many predators, as they provide cover, den sites, and a variety of different food resources (e.g., small mammals, bird nests, fruit) throughout the year (Janko et al., 2012; Keuling et al., 2011; Michel et al., 2007). Consequently, wood‐rich landscapes may support high fox numbers and increase fox activity in the surrounding areas.

Foxes are known to be synanthropic—they regularly use anthropogenic food sources and inhabit even large cities (Contesse et al., 2004; Duduś et al., 2014; Harris & Rayner, 1986; Jankowiak et al., 2008). Villages with surrounding gardens and small scale livestock and poultry farming, as in our study area, provide a variety of food sources for foxes, which could explain why the number of fox captures was higher closer to settlements (Janko et al., 2012; Jankowiak et al., 2008). Consequently, if villages attract foxes, predation risk by foxes is likely to decrease with increasing distance from settlements.

Interestingly, water surface area had a negative relationship with fox captures, in contrast to previous studies that showed some preference for water‐related habitats in foxes (Fiderer et al., 2019; Kuehl & Clark, 2002; Matos et al., 2009). In our study area, lakes and streams were generally surrounded by reed beds, hedges, and woods. This high availability of attractive vegetation structures may have led to a dilution effect, where predator activity near water was higher, but predators were more dispersed and less likely to pass the camera station.

These results suggest that the optimal landscape to reduce predation risk for ground‐nesting farmland birds would be open farmland with small field sizes and many edge structures, but little to no woods and settlements. Interestingly, several studies came to similar conclusions regarding the ideal landscape for farmland birds. Guerrero et al. (2012) concluded that farmland bird densities in several European countries were higher in landscapes dominated by agriculture with small fields and a high crop diversity. A recent cross‐border study in Austria and the Czech Republic also found a positive association between farmland bird abundance and diversity and habitat heterogeneity (Šálek et al., 2021). In Finland, field edge density had strong positive effects on farmland bird assemblages and seemed to be even more important than crop diversity, grassland, or fallows (Ekroos et al., 2019). These results are usually explained by a lack of nesting habitats and food resources in high intensity farmland compared to fallows, field margins, grasslands, and diverse crops (Ekroos et al., 2019; Guerrero et al., 2012; Šálek et al., 2021). Our results, however, suggest that predator activity may also play a role. If predator activity is lower or less dense in a landscape optimal for ground‐nesting farmland birds, we would expect lower predation rates and higher breeding success, and therefore higher bird densities.

## 5 CONCLUSION

By looking at the landscape from a (mammalian) predators' point of view, we can distinguish between intensively used areas and those with less predator activity that are consequently safer for ground‐nesting birds. Understanding what factors affect the distribution of predator activity allows us to adapt management plans to mitigate predation risk and improve nesting success.

In summary, our study shows that predator activity depended primarily on vegetation type and additionally on wood cover, landscape structure, distance to settlements, and habitat diversity. Flower strips were shown to provide less risky nesting habitat than other permanent vegetation structures such as hedges and field margins. Based on these results, several recommendations for the conservation of ground‐nesting farmland birds are possible: First, flower strips can be highly recommended as a conservation measure, as they provide not only good nesting habitat but also lower the predation risk compared to other permanent vegetation structures. Broad flower blocks should be preferred over narrow strips, because predator activity and predation risk is higher along the edges. Second, flower blocks and similar conservation measures for ground‐nesting birds should ideally be placed in areas with little wood cover and away from settlements wherever possible, because woods support high numbers of predators and settlements are attractive for generalist predators, leading to higher predator activity and higher predation risk close to these features. Third, highly structured landscapes seem to decrease predation risk by reducing the encounter probability between birds and predators. Therefore, small‐scale structures such as field margins, ditches, and fallows should be preserved and the use of small field sizes encouraged. The optimal landscape for ground‐nesting farmland birds seems to be open farmland with small fields, many edge structures, and broad flower blocks or similar areas as breeding habitat.

## AUTHOR CONTRIBUTIONS

**Amelie Laux:** Conceptualization (equal); data curation (lead); formal analysis (lead); funding acquisition (equal); investigation (lead); project administration (lead); visualization (lead); writing – original draft (lead). **Matthias Waltert:** Conceptualization (equal); funding acquisition (equal); supervision (supporting); writing – review and editing (equal). **Eckhard Gottschalk:** Conceptualization (equal); funding acquisition (equal); supervision (lead); writing – review and editing (equal).

## AUTHOR CONTRIBUTIONS

**Amelie Laux:** Conceptualization (equal); data curation (lead); formal analysis (lead); funding acquisition (equal); investigation (lead); project administration (lead); visualization (lead); writing – original draft (lead). **Matthias Waltert:** Conceptualization (equal); funding acquisition (equal); supervision (supporting); writing – review and editing (equal). **Eckhard Gottschalk:** Conceptualization (equal); funding acquisition (equal); supervision (lead); writing – review and editing (equal).

## CONFLICT OF INTEREST

The authors declare that there is no conflict of interests.

## Data Availability

All datasets and R scripts used in this study are available at the Dryad Digital Repository: https://doi.org/10.5061/dryad.1g1jwsv03.

## Appendix A

**TABLE A1:** Shapiro–Wilk normality test for each predator species and all predators. “all predators” includes all predator species except dogs

Season	Predator species	W	*p*‐value
2019	All predators	0.430	<.001
	Badger	0.468	<.001
	Boar	0.345	<.001
	Cats	0.244	<.001
	Dog	0.118	<.001
	Fox	0.577	<.001
	Marten	0.222	<.001
	Mouse weasel	0.065	<.001
	Racoon	0.126	<.001
	Stoat	0.108	<.001
2020	All predators	0.472	<.001
	Badger	0.287	<.001
	Boar	0.330	<.001
	Cats	0.296	<.001
	Dog	0.147	<.001
	Fox	0.245	<.001
	Marten	0.251	<.001
	Mouse weasel	0.065	<.001
	Racoon	0.214	<.001
	Stoat	0.137	<.001
2019 + 2020	All predators	0.451	<.001
	Badger	0.35	<.001
	Boar	0.331	<.001
	Cats	0.271	<.001
	Dog	0.126	<.001
	Fox	0.347	<.001
	Marten	0.234	<.001
	Mouse weasel	0.057	<.001
	Racoon	0.141	<.001
	Stoat	0.121	<.001

**TABLE A2:** Comparison of mean capture rates (captures/100 camera days) in all vegetation types between the areas Diemarden and Eichsfeld. Wilcoxon rank‐sum test with continuity correction. “all predators” includes all predator species except dogs. Years 2019 and 2020, N_Cameras_ (Diemarden) = 68, N_Cameras_ (Eichsfeld) = 176

Predator species	W	*p*‐value
All predators	6038	.694
Badger	5344	.231
Boar	5964.5	.74
Cats	5897	.835
Dog	6408	.006
Fox	5733	.809
Marten	5983	.543
Mouse weasel	5780	.376
Racoon	6329	.223
Stoat	5797	.674

**TABLE A3:** Comparison of mean capture rates (captures/100 camera days) between Years 2019 and 2020. Wilcoxon rank‐sum test with continuity correction. “all predators” includes all predator species except dogs. “‐“ marks species not found in the respective vegetation type. N_Cameras_(all vegetation types)= 120, N_Cameras_ (single vegetation types) = 24 in 2019 and 2020, respectively

Predator species	Vegetation type	W	*p*‐value
Field margin	All predators	317	.556
	Badger	375	.054
	Boar	311	.419
	Cats	300	.338
	Dog	284	.911
	Fox	286	.975
	Marten	287.5	1
	Mouse weasel	–	–
	Racoon	276	.699
	Stoat	301	.539
Flower strip	All predators	377.5	.065
	Badger	339	.171
	Boar	336.5	.085
	Cats	301	.627
	Dog	288.5	1
	Fox	312	.620
	Marten	–	–
	Mouse weasel	300	.338
	Racoon	303	.626
	Stoat	276	.338
Hedge	All predators	268	.688
	Badger	257.5	.509
	Boar	224	.075
	Cats	263.5	.446
	Dog	288	1
	Fox	300	.812
	Marten	283.5	.908
	Mouse weasel	276	.338
	Racoon	316	.558
	Stoat	276	.338
Rapeseed	All predators	287.5	1
	Badger	284	.939
	Boar	221	.131
	Cats	242.5	.106
	Dog	–	–
	Fox	325.5	.443
	Marten	284.5	.914
	Mouse weasel	–	–
	Racoon	250	.419
	Stoat	–	–
Winter cereal	All predators	272	.721
	Badger	276	.655
	Boar	290	.962
	Cats	–	–
	Dog	276	.338
	Fox	268.5	.626
	Marten	–	–
	Mouse weasel	–	–
	Racoon	277	.606
	Stoat	–	–
All vegetation types	All predators	7454.5	.635
	Badger	7438	.348
	Boar	6955	.528
	Cats	6971	.375
	Dog	7133	.768
	Fox	7322.5	.816
	Marten	7179.5	.935
	Mouse weasel	7200.5	1
	Racoon	7104	.827
	Stoat	7141	.66

**TABLE A4:** General Variance Inflation Factors for all predictors considered in the full models of model set 1

	GVIF	Degree of freedom	GVIF^(1/2Df)
Border_length	2.633	1	1.623
Edge_Area	2.081	1	1.443
Edge_Dist	3.752	1	1.937
Ext_Area	2.255	1	1.502
Habitat_diversity	1.697	1	1.303
Road_Dist	1.363	1	1.168
Settl_Area	1.600	1	1.265
Settl_Dist	1.773	1	1.332
Water_Area	1.455	1	1.206
Water_Dist	1.545	1	1.243
Wood_Area	1.309	1	1.144
Wood_Dist	1.864	1	1.365
Vegetation type	6.917	4	1.273

**TABLE A5:** Moran’s *I* test for spatial autocorrelation for raw data and model set 1 residuals. “all predators” includes all predator species except dogs. N_Cameras_(2019) = 120, N_Cameras_(2020) = 120. Models were fit with 2019 and 2020 data

Raw data	Predator	Season	Observed	Expected	SD	*p*‐value
	All predators	2019	−0.013	−0.008	0.012	.699
	All predators	2020	−0.022	−0.008	0.014	.337
	Fox	2019	0.011	−0.008	0.016	.209
	Fox	2020	−0.020	−0.008	0.008	.164
GLMM	Response variable	Model	Observed	Expected	SD	p‐value
	All predators	M1. full model	0.046	−0.004	0.064	.438
		M1. final model	0.07	−0.004	0.064	.249
	Fox	M1. full model	−0.014	−0.004	0.064	.873
		M1. final model	−0.039	−0.004	0.064	.583

**TABLE A6:** Runtime, number of predator observations and cameras with predator observations in both seasons. N_Cameras_(all vegetation types) = 120, N_Cameras_ (single vegetation types) = 24 in 2019 and 2020, respectively.

		Summer 2019	Summer 2020
	Runtime	2520.363 days	2504.334 days
	Mean runtime	21.00 days	20.87 days
Number of predator observations	Observations total	1099	1023
	Badger	146	142
	Boar	81	110
	Cat	17	20
	Dog	26	45
	Fox	489	460
	Marten	18	27
	Mouse weasel	2	1
	Racoon	318	205
	Stoat	2	4
Number of cameras with predator observation	Vegetation	Summer 2019	Summer 2020
	Field margin	20	20
	Flower strip	21	17
	Hedge	22	23
	Rapeseed	23	23
	Winter cereal	10	10

**TABLE A7:** Mean capture rates (captures/100 camera days) of all predators in each vegetation type. “all predators” includes all predator species except dogs. Years 2019 and 2020 together, N_Cameras_(all vegetation types) = 240, N_Cameras_ (single vegetation types) = 48. SD = standard deviation, CI = confidence interval

Vegetation type	Predator species	Mean capture rate	SD	95% CI
Field margins	All predators	26.655	35.507	10.310
	Badger	4.637	7.488	2.174
	Boar	3.635	17.835	5.179
	Cats	0.099	0.687	0.199
	Dog	7.273	25.793	7.489
	Fox	16.027	25.023	7.266
	Marten	0.281	1.448	0.420
	Mouse weasel	0.000	0.000	0.000
	Racoon	1.681	5.197	1.509
	Stoat	0.294	1.150	0.334
Flower strips	All predators	19.086	23.231	6.746
	Badger	2.214	4.761	1.383
	Boar	2.737	11.124	3.230
	Cats	1.154	3.858	1.120
	Dog	0.290	1.477	0.429
	Fox	10.356	15.035	4.366
	Marten	0.000	0.000	0.000
	Mouse weasel	0.261	1.805	0.524
	Racoon	2.274	7.651	2.222
	Stoat	0.091	0.631	0.183
Hedge	All predators	87.925	151.615	44.024
	Badger	12.059	30.959	8.990
	Boar	4.796	13.957	4.053
	Cats	1.466	4.194	1.218
	Dog	0.673	2.544	0.739
	Fox	33.177	93.584	27.174
	Marten	3.183	7.687	2.232
	Mouse weasel	0.095	0.658	0.191
	Racoon	32.959	120.515	34.994
	Stoat	0.190	1.316	0.382
Rapeseed	All predators	56.884	55.884	16.227
	Badger	8.470	13.173	3.825
	Boar	5.901	10.101	2.933
	Cats	0.975	3.208	0.931
	Dog	0.000	0.000	0.000
	Fox	30.182	50.327	14.614
	Marten	0.729	2.105	0.611
	Mouse weasel	0.000	0.000	0.000
	Racoon	10.627	24.860	7.219
	Stoat	0.000	0.000	0.000
Winter cereal	All predators	6.728	15.708	4.561
	Badger	0.747	2.443	0.709
	Boar	1.286	3.577	1.039
	Cats	0.000	0.000	0.000
	Dog	0.190	1.315	0.382
	Fox	4.331	13.606	3.951
	Marten	0.000	0.000	0.000
	Mouse weasel	0.000	0.000	0.000
	Racoon	0.364	1.493	0.433
	stoat	0.000	0.000	0.000
All vegetation types	All predators	39.456	80.009	10.174
	Badger	5.625	16.016	2.037
	Boar	3.671	12.262	1.559
	Cats	0.739	2.974	0.378
	Dog	1.685	11.864	1.509
	Fox	18.815	50.596	6.421
	Marten	0.839	3.789	0.482
	Mouse weasel	0.071	0.858	0.109
	Racoon	9.581	56.083	7.131
	Stoat	0.115	0.832	0.106

**TABLE A8:** Kruskal–Wallis rank sum test of predator capture rates (captures/100 camera days) within each vegetation type. Years 2019 and 2020 together, N_Cameras_(all vegetation types)= 240, N_Cameras_ (single vegetation types) = 48

Vegetation type	Kruskal Wallis *χ*²	Degrees of freedom	*p*‐value
Field margin	139.87	8	<.001
Flower strip	123.55	8	<.001
Hedge	145.45	8	<.001
Rapeseed	170.13	8	<.001
Winter cereal	58.348	8	<.001
All vegetation types	543.64	8	<.001

**TABLE A9:** Post Hoc Dunn’s Test comparison between predator capture rates (captures/100 camera days) within each vegetation type. Years 2019 and 2020 together, N_Cameras_(all vegetation types) = 240, N_Cameras_ (single vegetation types) = 48.

Comparison	All vegetation types		Field margin		Flower strips	
	Z‐statistic	Adjusted *p*‐value	Z‐statistic	Adjusted *p*‐value	Z‐statistic	Adjusted *p*‐value
Badger – boar	4.166	<.001	4.155	.001	1.547	1
Badger – cats	7.844	<.001	5.466	<.001	1.890	1
Boar – cats	3.678	.003	1.311	1	0.343	1
Badger – dog	8.290	<.001	3.556	.009	2.760	.145
Boar – dog	4.124	.001	−0.600	1	1.213	1
Cats – dog	0.445	1	−1.911	1	0.870	1
Badger – fox	−7.851	<.001	−3.084	.045	−5.597	<.001
Boar – fox	−12.017	<.001	−7.239	<.001	−7.144	<.001
Cats – fox	−15.696	<.001	−8.550	<.001	−7.487	<.001
Dog – fox	−16.141	<.001	−6.639	<.001	−8.357	<.001
Badger – marten	8.039	<.001	5.229	<.001	3.300	.027
Boar – marten	3.873	.002	1.074	1	1.753	1
Cats – marten	0.195	.846	−0.237	1	1.410	1
Dog – marten	−0.250	1	1.673	1	0.540	1
Fox – marten	15.890	<.001	8.312	<.001	8.897	<.001
Badger – mouse weasel	9.791	<.001	5.698	<.001	3.007	.069
Boar – mouse weasel	5.625	<.001	1.543	1	1.460	1
Cats – mouse weasel	1.946	.568	0.232	1	1.117	1
Dog – mouse weasel	1.501	.933	2.142	.675	0.247	1
Fox – mouse weasel	17.642	<.001	8.781	<.001	8.604	<.001
Marten – mouse weasel	1.751	.799	0.469	1	−0.293	1
Badger – racoon	1.673	.849	3.929	.002	1.285	1
Boar – racoon	−2.493	.152	−0.226	1	−0.262	1
Cats – racoon	−6.172	<.001	−1.537	1	−0.605	1
Dog – racoon	−6.617	<.001	0.374	1	−1.475	1
Fox – racoon	9.524	<.001	7.013	<.001	6.881	<.001
Marten – racoon	−6.366	<.001	−1.300	1	−2.015	1
Mouse weasel – racoon	−8.118	<.001	−1.769	1	−1.722	1
Badger – stoat	9.495	<.001	5.017	<.001	3.057	.060
Boar – stoat	5.329	<.001	0.861	1	1.510	1
Cats – stoat	1.651	.790	−0.449	1	1.167	1
Dog – stoat	1.206	1	1.461	1	0.297	1
Fox – stoat	17.346	<.001	8.100	<.001	8.654	<.001
Marten – stoat	1.456	.872	−0.212	.832	−0.243	1
Mouse weasel – stoat	−0.295	1	−0.681	1	0.050	.960
Racoon – stoat	7.822	<.001	1.087	1	1.772	1

Comparison	Hedge		Rapeseed		Winter cereal	
	*Z*‐statistic	Adjusted *p*‐value	*Z*‐statistic	Adjusted *p*‐value	*Z*‐statistic	Adjusted *p*‐value
Badger – boar	2.942	.059	0.968	1	−1.167	1
Badger – cats	3.721	.004	4.329	<.001	2.000	1
Boar – cats	4.495	1	3.361	.012	3.166	.045
Badger – dog	−3.292	<.001	5.564	<.001	1.592	1
Boar – dog	3.099	1	4.596	<.001	2.759	.145
Cats – dog	5.117	1	1.235	1	−0.407	1
Badger – fox	−1.258	.020	−3.255	.017	−3.502	.014
Boar – fox	5.084	<.001	−4.223	<.001	−2.336	.468
Cats – fox	0.779	<.001	−7.584	<.001	−5.502	<.001
Dog – fox	1.553	<.001	−8.819	<.001	−5.095	<.001
Badger – marten	−6.233	.037	4.422	<.001	2.000	.956
Boar – marten	0.158	1	3.454	.009	3.166	.043
Cats – marten	2.176	1	0.093	1	0	1
Dog – marten	−4.200	1	−1.142	1	0.407	1
Fox – marten	2.143	<.001	7.677	<.001	5.502	<.001
Badger – mouse weasel	0.774	<.001	5.564	<.001	2.000	.911
Boar – mouse weasel	−7.013	.503	4.596	<.001	3.166	.042
Cats – mouse weasel	−0.622	1	1.235	1	0	1
Dog – mouse weasel	1.396	1	0	1	0.407	1
Fox – mouse weasel	−4.979	<.001	8.819	<.001	5.502	<.001
Marten – mouse weasel	1.363	.610	1.142	1	0	1
Badger – racoon	−7.786	1	−0.357	1	0.841	1
Boar – racoon	−1.395	.001	−1.325	1	2.008	1
Cats – racoon	0.623	<.001	−4.685	<.001	−1.158	1
Dog – racoon	−5.753	<.001	−5.920	<.001	−0.751	1
Fox – racoon	0.590	.630	2.898	.053	4.344	<.001
Marten – racoon	6.391	<.001	−4.779	<.001	−1.158	1
Mouse weasel – racoon	8.409	<.001	−5.920	<.001	−1.158	1
Badger – stoat	2.033	<.001	5.564	<.001	2.000	.865
Boar – stoat	8.376	.514	4.596	<.001	3.166	.040
Cats – stoat	2.018	1	1.235	1	0	1
Dog – stoat	−4.358	1	0	1	0.407	1
Fox – stoat	1.985	<.001	8.819	<.001	5.502	<.001
Marten – stoat	−6.376	.613	1.142	1	0	1
Mouse weasel – stoat	−0.033	.974	0	1	0	1
Racoon – stoat	6.343	<.001	5.920	<.001	1.158	1

**TABLE A10:** Kruskal–Wallis rank sum test of predator capture rates (captures/100 camera days) between vegetation types. “all predators” includes all predator species except dogs. Years 2019 and 2020 together, N_Cameras_ = 48 in each vegetation type

Predator species	Kruskal Wallis *χ*²	Degrees of freedom	*p*‐value
All predators	78.308	4	<.001
Badger	29.887	4	<.001
Boar	18.527	4	<.001
Cats	12.454	4	.014
Fox	37.348	4	<.001
Marten	22.937	4	<.001
Mouse weasel	3.013	4	.556
Racoon	61.155	4	<.001
Stoat	6.102	4	.192

**TABLE A11:** Post Hoc Dunn’s Test comparison between predator capture rates (captures/100 camera days) between vegetation types for each predator species and all predators. “all predators” includes all predator species except dogs. Years 2019 and 2020 together, N_Cameras_ = 48 in each vegetation type

Comparison	All predators		Badger		Boar	
	*z*‐value	Adjusted *p*‐value	*z*‐value	Adjusted *p*‐value	*z*‐value	Adjusted *p*‐value
Field margin – hedge	−3.588	.002	−0.852	1	−1.192	1
Field margin – rapeseed	−3.121	.005	−0.835	.808	−3.640	.003
Field margin – winter cereal	4.126	<.001	3.484	.004	−0.386	1
Flower strip – field margin	−0.570	1	−2.104	.177	0.055	.956
Flower strip – hedge	−4.157	<.001	−2.955	.022	−1.137	1
Flower strip – rapeseed	−3.691	.001	−2.938	.020	−3.585	.003
Flower strip – winter cereal	3.557	.002	1.381	.669	−0.330	1
Hedge – rapeseed	0.466	.641	0.017	.986	−2.448	.101
Winter cereal – hedge	−7.714	<.001	−4.336	<.001	−0.807	1
Winter cereal – rapeseed	−7.248	<.001	−4.319	<.001	−3.255	.009
Comparison	Cats		Fox		Marten	
	Z‐statistic	Adjusted *p*‐value	Z‐statistic	Adjusted *p*‐value	Z‐statistic	Adjusted *p*‐value
Field margin – hedge	−2.572	.091	−1.018	1	−3.168	.012
Field margin – rapeseed	−1.848	.388	−0.972	.994	−1.497	.538
Field margin – winter cereal	0.360	1	4.235	<.001	0.763	.891
Flower strip – field margin	1.500	.668	−0.724	.938	−0.763	1
Flower strip – hedge	−1.072	1	−1.742	.489	−3.931	.001
Flower strip – rapeseed	−0.348	.728	−1.696	.450	−2.260	.167
Flower strip – winter cereal	1.860	.440	3.511	.003	<0.001	1
Hedge – rapeseed	0.724	1	0.047	.963	1.671	.474
Winter cereal – hedge	−2.932	.034	−5.253	<.001	−3.931	.001
Winter cereal – rapeseed	−2.208	.218	−5.206	<.001	−2.260	.143
Comparison	Mouse weasel		Racoon		Stoat	
	Z‐statistic	Adjusted *p*‐value	Z‐statistic	Adjusted *p*‐value	Z‐statistic	Adjusted *p*‐value
Field margin – hedge	−1.116	1	−5.152	<.001	1.414	1
Field margin – rapeseed	0	1	−4.230	<.001	2.139	.292
Comparison	Mouse weasel		Racoon		Stoat	
	Z‐statistic	Adjusted *p*‐value	Z‐statistic	Adjusted *p*‐value	Z‐statistic	Adjusted *p*‐value
Field margin – winter cereal	0	1	0.899	.737	2.139	.324
Flower strip – field margin	1.125	1	0.083	.934	−1.438	1
Flower strip – hedge	0.009	1	−5.069	<.001	−0.024	1
Flower strip – rapeseed	1.125	1	−4.147	<.001	0.701	1
Flower strip – winter cereal	1.125	1	0.982	1	0.701	1
Hedge – rapeseed	1.116	1	0.923	1	0.725	1
Winter cereal – hedge	−1.116	1	−6.051	<.001	−0.725	1
Winter cereal – rapeseed	0	1	−5.129	<.001	0	1

**TABLE A12:** Model results of M1 Predator activity, full model Negative binomial general linear mixed model. For variable abbreviations see table 1. N_Cameras_ = 240. SE = standard error. SD = standard deviation. AICc = 1387.24. Conditional *R*² = 0.577. Marginal *R*² = 0.544. Dispersion parameter = 0.896

Fixed effects					
Predictors	Estimates	SE	*z*‐value	*p*‐value	Relative importance
Intercept	−8.889	0.9	−9.888	<.001	
Border_Length	−0.016	0.045	−0.353	.724	0.365
Edge_Area	−0.256	0.13	−1.961	.05	6.860
Edge_Dist	0.005	0.004	1.258	.208	5.220
Ext_Area	−0.014	0.018	−0.776	.438	1.404
Hab_Div	0.956	0.351	2.723	.006	9.157
Road_Dist	0.000	0.000	−1.025	.305	1.365
Settl_Area	−0.053	0.039	−1.374	.17	3.990
Settl_Dist	0.000	0.000	−1.087	.277	1.886
Water_Area	−0.219	0.094	−2.334	.02	9.255
Water_Dist	0.000	0.000	−0.696	.486	0.853
Wood_Area	0.028	0.02	1.373	.17	5.412
Wood_Dist	−0.001	0.001	−1.074	.283	1.844
Vegetation – field margin	0.615	0.308	1.999	.046	Vegetation type 52.387
Vegetation – hedge	1.607	0.304	5.293	<.001	
Vegetation – rapeseed	0.899	0.304	2.954	.003	
Vegetation – winter cereal	−1.328	0.356	−3.73	<.001	
Random effects					
Predictors	Variance	SD	Groups	N_Observations_	
Season:Block	0.064	0.252	8	240	

**TABLE A13:** Model results of M1 Predator activity after backward selection. Negative binomial generalized linear mixed model. For variable abbreviations see table 1. SE = standard error, SD = standard deviation. AICc = 1376.548, Conditional *R*² = 0.557, Marginal *R*² = 0.521. Dispersion parameter = 0.867

Fixed effects							
Predictors		Estimates	SE	*z*−value	*p*−value	Odds ratio	Relative importance
Intercept		−9.746	0.497	−19.627	<.001		
Water_Area		−0.156	0.081	−1.914	.056	0.856	6.259
Edge_Area		−0.174	0.103	−1.687	.092	0.840	4.557
Hab_Div		0.766	0.3	2.553	.011	2.152	9.589
Wood_Area		0.027	0.018	1.49	.136	1.028	6.415
Vegetation	Field margin	0.557	0.27	2.064	.039	1.746	Vegetation type
	Winter cereal	−1.025	0.293	−3.494	<.001	0.359	73.179
	Hedge	1.573	0.253	6.211	<.001	4.819	
	Rapeseed	1.137	0.251	4.541	<.001	3.119	
Random effects							
		Variance	SD	Groups	N_Observations_		
Year:Block		0.066	0.257	8	240		

**TABLE A14:** Model results of M1 Fox activity, full model. Negative binomial general linear mixed model. For variable abbreviations see table 1. N_Cameras_ = 240. SE = standard error. SD = standard deviation. AICc = 1080.689. Conditional *R*² = 0.45. Marginal *R*² = 0.434. Dispersion parameter = 0.542

Fixed effects					
Predictors	Estimates	SE	*z*‐value	*p*‐value	Relative importance
Intercept	−8.823	1.222	−7.221	<.001	
Border_Length	−0.073	0.062	−1.177	.239	6.144
Edge_Area	−0.09	0.169	−0.534	.594	0.894
Edge_Dist	0.006	0.005	1.2	.23	10.842
Ext_Area	−0.037	0.022	−1.698	.089	7.202
Hab_Div	0.827	0.476	1.737	.082	7.155
Road_Dist	0.000	0.000	−0.76	.447	1.432
Settl_Area	−0.041	0.057	−0.718	.473	3.209
Settl_Dist	−0.001	0.001	−1.205	.228	4.122
Water_Area	−0.224	0.126	−1.783	.075	6.198
Water_Dist	0.000	0.000	0.708	.479	2.076
Wood_Area	0.027	0.026	1.038	.299	5.762
Wood_Dist	−0.002	0.002	−1.021	.307	3.015
Vegetation – field margin	0.336	0.411	0.817	.414	Vegetation type
Vegetation – hedge	0.963	0.414	2.325	.02	41.948
Vegetation – rapeseed	0.565	0.38	1.486	.137	
Vegetation – winter cereal	−1.703	0.484	−3.52	<.001	
Random effects					
Predictors	Variance	SD	Groups	N_Observations_	
Season:Block	0.032	0.179	8	240	

**TABLE A15:** Comparison of “all predator” and fox capture rates (captures/100 camera days) in flower strips (edge and centre together) between the areas Diemarden and Nesselröden. Wilcoxon rank‐sum test with continuity correction. “all predators” includes all predator species except dogs. At edge cameras, only predators that passed within 1m of the camera were included. N_Cameras_ (Diemarden) = 24, N_Cameras_ (Eichsfeld) = 24

Predator species	W	*p*‐value
All predators	230	.232
Fox	252	.452

**TABLE A16:** Mean capture rates (captures/100 camera days) of all predators in the centre and at the edge of flower strips “all predators” includes all predator species except dogs. “–“ marks species not found in the respective vegetation type. N_Cameras_ = 24 at each position. At edge cameras, only predators that passed within 1m of the camera were included. Additionally, capture rates and observations of all predators at the edge regardless of the distance to the camera are given below. SD = standard deviation, CI = confidence interval

	Predator species	Mean capture rate	SD	95% CI	Observations	Cameras with observations
Centre	All predators	5.062	6.047	2.554	27	13
	Badger	0.926	2.251	0.951	5	4
	Boar	0.371	1.256	0.530	2	2
	Cats	–	–	–	–	0
	Dog	–	–	–	–	0
	Fox	2.447	3.703	1.563	13	9
	Marten	–	–	–	–	0
	Mouse weasel	0.954	3.812	1.610	5	2
	Racoon	0.364	1.233	0.521	2	2
Edge	All predators	49.240	42.839	18.089	193	23
	Badger	7.140	10.966	4.631	33	11
	Boar	4.620	15.495	6.543	17	5
	Cats	8.232	19.487	8.229	19	8
	Dog	4.009	9.732	4.109	17	6
	Fox	22.896	22.297	9.415	97	21
	Marten	0.182	0.889	0.375	1	1
	Mouse weasel	0.183	0.897	0.379	1	1
	Racoon	6.242	13.682	5.777	19	6
Edge all captures	All predators	60.985	53.312	22.512	234	23
	Badger	8.408	12.167	5.138	38	12
	Boar	5.319	18.842	7.956	19	5
	Cats	8.232	19.487	8.229	19	8
	Dog	4.009	9.732	4.109	17	6
	Fox	31.468	34.525	14.579	127	21
	Marten	0.182	0.889	0.375	1	1
	Mouse weasel	0.183	0.897	0.379	1	1
	Racoon	6.242	13.682	5.777	23	6

**TABLE A17:** Kruskal–Wallis rank sum test of predator capture rates (captures/100 camera days) in the centre and at the edge of flower strips. At edge cameras, only predators that passed within 1 m of the camera were included. N_Cameras_ (centre) = 24, N_Cameras_ (edge) = 24

Position	Kruskal Wallis χ²	Degrees of freedom	*p*‐value
Centre	29.967	7	<.001
Edge	61.931	7	<.001

**TABLE A18:** Post Hoc Dunn’s Test comparison between predator capture rates (captures/100 camera days) in the centre and at the edge of flower strips. “all predators” includes all predator species except dogs. “–“ marks species not found in the respective vegetation type. At edge cameras, only predators that passed within 1m of the camera were included. N_Cameras_ = 24 at each position

Comparison	Centre		Edge	
	Z‐statistic	Adjusted *p*‐value	Z‐statistic	Adjusted *p*‐value
Badger – boar	1.018	1	1.771	1
Badger – cats	1.940	1	0.881	1
Badger – dog	1.940	1	1.521	1
Badger – fox	−2.414	.347	−3.549	.008
Badger – marten	1.940	.995	3.041	.05
Badger – mouseweasel	0.917	1	3.026	.05
Badger – racoon	1.035	1	1.346	1
Boar – cats	0.922	1	−0.890	1
Boar – dog	0.922	1	−0.250	1
Boar – fox	−3.431	.014	−5.320	<.001
Boar – marten	0.922	1	1.270	1
Boar – mouseweasel	−0.100	1	1.256	1
Boar – racoon	0.018	1	−0.424	1
Cats – dog	0.000	1	0.641	1
Cats – fox	−4.354	<.001	−4.430	<.001
Cats – marten	0.000	1	2.160	.585
Cats – mouseweasel	−1.023	1	2.146	.574
Cats – racoon	−0.905	1	0.466	1
Dog – fox	−4.354	<.001	−5.070	<.001
Dog – marten	0.000	1	1.520	1
Dog – mouseweasel	−1.023	1	1.505	1
Dog – racoon	−0.905	1	−0.175	1
Fox – marten	4.354	<.001	6.59	<.001
Fox – mouseweasel	3.331	.020	6.576	<.001
Fox – racoon	3.449	.014	4.896	<.001
Marten – mouseweasel	−1.023	1	−0.014	.989
Marten – racoon	−0.905	1	−1.694	1
Mouseweasel – racoon	0.118	1	−1.680	1

**TABLE A19:** Comparison of mean capture rates (captures/100 camera days) between the edge and the centre of flower strips. Wilcoxon signed rank test with continuity correction. “all predators” includes all predator species except dogs. At edge cameras, only predators that passed within 1m of the camera were included. N_Cameras_ (Diemarden) = 24, N_Cameras_ (Eichsfeld) = 24

Predator species	V	*p*‐value
All predators	13	<.001
Fox	15	<.001
Badger	8	.017
Boar	3	.076
Racoon	1	.035
Marten	0	1
Cats	0	.014
Dog	0	.036
Mouse weasel	5	.423

## Appendix B

### B.1 Model set M2: Comparison of predator and fox activity at different scales

To investigate how the effects of landscape composition on predator activity differ on different scales, we constructed three different GLMMs based on predictors measured in 500 m, 1 km and 2.5 km buffers around the camera sites. We used only the main land use types as predictors for these models, because detailed data of small vegetation structures was not available on larger scales.

### B.2 Environmental predictors and study covariates

Table B1 shows the predictors that were considered at each scale. Measurements were calculated in R (R Core Team, 2021) based on B‐DLM (LGLN 2019; TLBG 2019) and InVeKos maps (SLA 2019b, SLA 2019a, SLA 2020). After calculating GVIFs for the predictors at each scale, arable land was dropped in all cases, as its area is closely related to forest area (Table B2). Time block nested in year was included as a random effect to account for temporal variation in predator activity.

**TABLE B1:** List of predictors considered in the analysis of predator and fox activity in model set 2. Land cover predictors were measured within three different buffers of 500 m, 1 km and 2.5 km. Predictors in grey were not used in the full models due to collinearity issues. Data sources: B‐DLM (LGLN 2019; TLBG 2019), InVeKos (SLA 2019b, SLA 2019a, SLA 2020), our maps

	Predictor	Explanation	Unit	Source
Land cover	Forest_Area	Area of forests	ha	B‐DLM
	Grass_Area	Area of grassland	ha	InVeKos
	Arable_Area	Area of arable land	ha	Invekos
	Settl_Area	Area of settlements	ha	B‐DLM
	Water_Area	Surface area of all running and standing water	ha	B‐DLM
Site based	Vegetation	Vegetation type at camera site: Field margin, flower strip, hedge, rapeseed or winter cereal	factor	Empirical
	Year	2019 or 2020	factor	Empirical
	Block	Time blocks 1‐4 in each year	factor	Empirical
	Runtime	Active camera time	min	Empirical

**TABLE B2:** General Variance Inflation Factors for all predictors considered in the full models of model set 2

Scale	Predictor	GVIF	Df	GVIF^(1/2Df)
500 m	Grass_Area 500 m	1.177	1	1.085
	Settl_Area 500 m	1.069	1	1.034
	Water_Area 500 m	1.108	1	1.052
	Wood_Area 500 m	1.044	1	1.022
	Vegetation type	1.272	4	1.031
1 km	Grass_Area 1 km	1.084	1	1.041
	Settl_Area 1 km	1.057	1	1.028
	Water_Area 1 km	1.092	1	1.045
	Wood_Area 1 km	1.051	1	1.025
	Vegetation type	1.119	4	1.014
2.5 km	Grass_Area 2.5 km	1.252	1	1.119
	Settl_Area 2.5 km	1.063	1	1.031
	Water_Area 2.5 km	1.411	1	1.188
	Wood_Area 2.5 km	1.220	1	1.104
	Vegetation type	1.136	4	1.016

### B.3 Model formulation and relative variable importance

Separate models were fit for “all predators” and “fox” as response variables using the same procedure as described for model set 1. Predictors were measured within 500 m, 1 km or 2.5 km, respectively. A negative binomial regression was used, because a Poisson distribution resulted in high overdispersion and a bad model fit. There was no improvement with a zero‐inflated negative binomial distribution. All models included forest area, grassland area, water area, settlement area and vegetation as fixed effects and time block nested in year as random effect, with the runtime of each camera as offset. Full models are reported. AICc values for all models were compared using the bbmle – package (Bolker, R Development Core Team and Giné‐Vázquez, 2021). For each model the relative variable importance was calculated as described for model set 1.

**TABLE B3:** Moran’s *I* test for spatial autocorrelation for model set 2 residuals. “all predators” includes all predator species except dogs. N_Cameras_(2019) = 120, N_Cameras_(2020) = 120. Models were fit with 2019 and 2020 data

Response variable	Model	Observed	Expected	SD	*p*‐value
All predators	M2. 500 m	0.081	−0.004	0.064	.18
	M2. 1 km	0.100	−0.004	0.064	.103
	M2. 2.5 km	0.034	−0.004	0.064	.551
Fox	M2. 500 m	0.056	−0.004	0.064	.348
	M2. 1 km	0.032	−0.004	0.064	.575
	M2. 2.5 km	0.121	−0.004	0.064	.050

**TABLE B4:** Model results of M2 Predator activity models at different scales. Negative binomial general linear mixed models. For variable abbreviations see Table B1. N_Cameras_ = 240. SE = standard error. SD = standard deviation

Predictors	Estimates	SE	*z*‐value	*p*‐value	Relative importance
(a) Model M2 Predator activity at 500 m					
AICc = 1380.336. Conditional *R*² = 0.557. Marginal *R*² = 0.53; dispersion parameter = 0.839					
Fixed effects					
Intercept	‐9.102	0.234	‐38.958	<.001	
Forest_Area 500 m	0.056	0.02	2.714	.007	32.738
Grass_Area 500 m	0.014	0.014	0.977	.329	1.045
Settl_Area 500 m	0.003	0.034	0.087	.931	0.019
Water_Area 500 m	‐0.132	0.082	‐1.613	.107	3.304
Vegetation–field margin	0.334	0.264	1.266	.206	Vegetation type
Vegetation – hedge	1.65	0.257	6.426	<.001	62.894
Vegetation – rapeseed	1.1	0.257	4.28	<.001	
Vegetation‐winter cereal	‐1.155	0.295	‐3.918	<.001	
Random effects					
	Variance	SD	Groups	N _Observations_	
Season:Block	0.053	0.229	8	240	
(b) Model M2 Predator activity at 1 km					
AICc = 1389.259. Conditional *R*² = 0.52. Marginal *R*² = 0.477; dispersion parameter = 0.819					
Fixed effects					
Intercept	‐9.048	0.264	‐34.294	<.001	
Forest_Area 1 km	0.002	0.003	0.629	.529	0.829
Grass_Area 1 km	‐0.002	0.006	‐0.299	.765	0.156
Settl_Area 1 km	0.004	0.005	0.752	.452	1.536
Water_Area 1 km	‐0.008	0.014	‐0.592	.554	0.686
Vegetation–field margin	0.369	0.264	1.394	.163	Vegetation type
Vegetation – hedge	1.634	0.259	6.31	<.001	96.793
Vegetation – rapeseed	1.178	0.26	4.531	<.001	
Vegetation‐winter cereal	‐0.989	0.287	‐3.453	.001	
Random effects					
	Variance	SD	Groups	N_Observations_	
Season:Block	0.076	0.276	8	240	
(c) Model M2 Predator activity at 2.5 km					
AICc = 1386.893. Conditional *R*² = 0.524. Marginal *R*² = 0.48; dispersion parameter = 0.83					
Fixed effects					
Intercept	‐8.677	0.419	‐20.721	<.001	
Forest_Area 2.5 km	0	0	0.417	.677	0.394
Grass_Area 2.5 km	‐0.001	0.002	‐0.358	.72	0.299
Settl_Area 2.5 km	‐0.001	0.001	‐1.837	.066	4.595
Water_Area 2.5 km	0	0.004	0.098	.922	0.024
Vegetation‐ field margin	0.368	0.262	1.404	.16	Vegetation type
Vegetation – hedge	1.559	0.257	6.067	<.001	94.689
Vegetation – rapeseed	1.179	0.26	4.535	<.001	
Vegetation‐winter cereal	‐1.064	0.288	‐3.679	<.001	
Random effects					
	Variance	SD	Groups	N_Observations_	
Season:Block	0.078	0.28	8	240	

**TABLE B5:** Model results of M2 Fox activity models at different scales. Negative binomial general linear mixed models. For variable abbreviations see Table B1. N_Cameras_ = 240. SE = standard error. SD = standard deviation

Predictors	Estimates	SE	*z*‐value	*p*‐value	Relative importance
(a) Model M2 Fox activity at 500 m					
AICc = 1069.981. Conditional *R*² = 0.439. Marginal *R*² = 0.435; dispersion parameter = 0.512					
Fixed effects					
Intercept	‐9.452	0.29	‐32.572	<.001	
Forest_Area 500 m	0.074	0.026	2.84	.005	61.707
Grass_Area 500 m	‐0.031	0.019	‐1.666	.096	2.261
Settl_Area 500 m	0.048	0.049	0.986	.324	2.648
Water_Area 500 m	‐0.214	0.102	‐2.095	.036	2.444
Vegetation – field margin	0.228	0.345	0.661	.509	Vegetation type
Vegetation – hedge	1.283	0.331	3.88	<.001	30.940
Vegetation – rapeseed	0.921	0.321	2.871	.004	
Vegetation – winter cereal	‐1.311	0.378	‐3.469	.001	
Random effects					
	Variance	SD	Groups	N_Observations_	
Season:Block	0.007	0.084	8	240	
(b) Model M2 Fox activity at 1 km					
AICc = 1078.713. Conditional *R*² = 0.348. Marginal *R*² = 0.340; dispersion parameter = 0.499					
Fixed effects					
Intercept	‐9.341	0.303	‐30.84	<.001	
Forest_Area 1 km	0.002	0.004	0.493	.622	1.113
Grass_Area 1 km	‐0.019	0.008	‐2.559	.011	16.963
Settl_Area 1 km	0.008	0.007	1.192	.233	7.099
Water_Area 1 km	‐0.005	0.019	‐0.264	.792	0.232
Vegetation – field margin	0.453	0.34	1.334	.182	Vegetation type
Vegetation – hedge	1.172	0.335	3.496	<.001	74.594
Vegetation – rapeseed	1.061	0.322	3.301	.001	
Vegetation – winter cereal	‐0.87	0.354	‐2.456	.014	
Random effects					
	Variance	SD	Groups	N_Observations_	
Season:Block	0.015	0.124	8	240	
(c) Model M2 Fox activity at 2.5 km					
AICc = 1075.676. Conditional *R*² = 0.363. Marginal *R*² = 0.359; dispersion parameter = 0.503					
Fixed effects					
Intercept	‐8.203	0.509	‐16.124	<.001	
Forest_Area 2.5 km	0	0.001	‐0.494	.622	0.939
Grass_Area 2.5 km	‐0.008	0.003	‐2.343	.019	22.663
Settl_Area 2.5 km	‐0.002	0.001	‐1.932	.053	8.712
Water_Area 2.5 km	0.003	0.005	0.595	.552	1.290
Vegetation – field margin	0.559	0.337	1.655	.098	Vegetation type
Vegetation – hedge	0.895	0.34	2.634	.008	66.396
Vegetation – rapeseed	1.104	0.326	3.384	.001	
Vegetation – winter cereal	‐1.025	0.358	‐2.867	.004	
Random effects					
	Variance	SD	Groups	N_Observations_	
Season:Block	0.007	0.081	8	240	

**TABLE B6:** Comparison of model AICc for M2 models of predator and fox activity on different scales

Model	Scale	AICc	ΔAICc	Degrees of freedom
Predator activity	500 m	1380.336	0.0	11
	1 km	1389.259	8.9	11
	2.5 km	1386.893	6.6	11
Fox activity	500 m	1069.981	0.0	11
	1 km	1078.713	8.7	11
	2.5 km	1075.676	5.7	11
